# Intensive Care Unit Admission for Pandemic (H1N1) 2009, Reunion Island, 2009

**DOI:** 10.3201/eid1701.100467

**Published:** 2011-01

**Authors:** Bernard-Alex Gaüzère, Denis Malvy, Laurent Filleul, Duksha Ramful, Marie-Christine Jaffar-Bandjee, Mounir El Bock, Khaled Ezzedine, David Vandroux

**Affiliations:** Author affiliations: Centre Hospitalier Régional de la Réunion (Saint-Pierre), Saint-Denis, France (B.-A. Gaüzère, D. Ramful, M.-C. Jaffar-Bandjee, D. Vandroux);; Université Bordeaux 2, Bordeaux, France (D. Malvy, K. Ezzedine);; Cellule de l’Institut de Veille Sanitaire en Eégion, Saint Denis (L. Filleul);; Centre Hospitalier Régional de la Réunion, Saint-Benoît, France (M. El Bock)

**Keywords:** H1N1 virus, intensive care units, Indian Ocean, La Réunion, influenza, viruses, pandemic, letter

**To the Editor:** We report results of the prospective surveillance system established in the largest intensive care unit (ICU) of Reunion Island (25 beds). This system covers 500,000 residents (62% of the total population) and monitors the daily status of patients >17 years of age who had a positive reverse transcription–PCR (RT-PCR) for pandemic (H1N1) 2009 virus. Reunion Island is a French overseas territory in the Southern Hemisphere, with health care facilities similar to those of mainland France. Patients were followed up until discharge from the ICU or death. Data were collected during July 15–September 30, 2009.

Of 148 patients with confirmed pandemic (H1N1) 2009 infection admitted to the hospital, 13 (9%) patients (8 female) were admitted to the ICU. These corresponded to 7% of all 187 patients admitted to the ICU during the same period. Median age was 39.4 (±19) years (range 17–69 years). Ten patients were admitted for respiratory failure related to viral pneumonitis, 1 for pulmonary edema with severe chronic coronary insufficiency, 1 for congenital adrenal insufficiency with reversible multiple organ failure, and 1 for status epilepticus. Eleven (85%) patients had underlying concurrent medical conditions: 3 were overweight (body mass indexes 38, 32, and 29.3 kg/m^2^); 1 was pregnant and had asthma.

Four (31%) patients died. One was a 28-year-old woman with cerebral motor infirmity and severe chronic restrictive respiratory failure. An 18-year-old woman with aplasia after receipt of an allograft for Hodgkin lymphoma died of cerebral hemorrhage while receiving extracorporeal membrane oxygenation. A 52-year-old man admitted for pulmonary edema with severe coronary insufficiency died of multiple organ failure. A 33-year-old man with no known concurrent medical conditions died of acute respiratory distress syndrome. Time from ICU admission to death ranged from 15 to 85 days (mean 36.5 ± 32 days). Mean age of patients who died was 32.5 ± 14.3 years.

Chest radiographic findings were abnormal for all patients except 1, who was admitted for fever and convulsions (Huntington chorea). Bilateral pulmonary embolism was confirmed in an obese patient who survived.

Mean time between onset of clinical signs and ICU admission was 6.9 ± 3.2 days. Mean time between admission to ICU with diagnosis confirmed by RT-PCR and initiation of antiviral treatment was 1.8 ± 1.7 days and between illness onset and initiation of antiviral treatment, 8.8 ± 3 days (range 4–16 days). Mean length of ICU stay was 26.3 ± 29.3 days. Patients remained in the ICU for a total of 201 bed-days (402 per million residents). The maximum daily occupancy of the ICU was 10 beds per million residents.

Five patients received steroids for severe hypotension or asthma-like clinical illness. Severe hypotension developed in 5 patients, and they received vasopressors. No patient received intravenous immunoglobulins. Ten (77%) patients required mechanical ventilation for a median of 11.5 ± 12.2 days. One patient required high-frequency ventilation, 3 required extracorporeal membrane oxygenation, and 1 required hemodialysis. Multiple organ failure developed in 3. All patients were empirically given antibacterial drugs. Secondary infections were either documented or strongly suspected for 5 patients.

All patients received oral oseltamivir beginning 4–16 days after illness onset and continuing for 2–17 days (mean 7.2 ± 4.3). Zanamivir was administered 1 time by inhalation through the ventilator. Viral loads in respiratory specimens ranged from 4 × 10^3^ to 6.9 × 10^7^ copies/mL (mean 1.4 × 10^5^). Two patients excreted virus in their bronchoalveolar lavage specimens for a prolonged time (14 days).

The most prominent biological findings were elevated serum levels of procalcitonin, C-reactive protein, aspartate aminotransferase, alanine aminotransferase, lactate dehydrogenase, and creatine kinase. Eight patients had lymphopenia (<1,200 cells/mm^3^).

Our findings are consistent with findings of other studies of severe or fatal viral pneumonia in younger patients than are usually affected in a normal influenza season (*1**–**4*), particularly in patients with concurrent medical conditions. In our study, the 3 overweight patients survived. Obesity is associated with increased severity of illness, but not always with death, in critically ill patients (*5*). We confirm that previously healthy young persons can die of pandemic (H1N1) 2009, although at a much lower rate than those infected in the initial outbreaks in Mexico (*6*) and the United States (*7*). In the United States, several pregnant women died, and the hospitalization rate for pregnant women was 4× higher than for the general population (*8*). Despite a fairly high birth rate on Reunion Island (19 births/1,000 population), our small series does not support these findings.

During the epidemic (July 20–September 20, 2009), acute respiratory infections, including presumed cases of pandemic (H1N1) 2009, accounted for 20.6% of the total case load of physicians on the island. The attack rate was ≈12.9% among the 810,000 inhabitants, and 8 deaths among persons with confirmed infection were reported. Therefore, the minimal overall death rate was ≈7.5 per million population and the case-fatality rate, 1 per 10,000 population.
